# Design and Manufacture of 30-Degree Projection Lens for Augmented Reality Waveguide

**DOI:** 10.3390/mi15101198

**Published:** 2024-09-27

**Authors:** Wen-Shing Sun, Ying-Shun Hsu, Chuen-Lin Tien, Wen-Kai Lin, Yi-Lun Su, Jun-Yi Yu, Shao-Kui Zhou, Yuan-Yan Liang, Wan-Pin Tsai, Chi Sun, Tsung-Xian Lee, Wei-Chia Su, Shiuan-Huei Lin, Ching-Cherng Sun

**Affiliations:** 1 Department of Optics and Photonics, National Central University, Chungli 32001, Taiwan; wssun@dop.ncu.edu.tw (W.-S.S.); dmcandymurray@yahoo.com.tw (Y.-S.H.); alan0734@gmail.com (W.-K.L.); a0357841@gmail.com (Y.-L.S.); 110226034@cc.ncu.edu.tw (J.-Y.Y.); ccsun65298@gmail.com (C.-C.S.); 2 Department of Electrical Engineering, Feng Chia University, Taichung 40724, Taiwan; 3 College of Photonics, National Yang Ming Chiao Tung University, Tainan 71150, Taiwan; tommychou848484@gmail.com (S.-K.Z.); m11125001@mail.ntust.edu.tw (W.-P.T.); albertsun@gmail.com (C.S.); lin@nycu.edu.tw (S.-H.L.); 4 Graduate Institute of Photonics, National Changhua University of Education, Changhua 50007, Taiwan; eri61206@gmail.com (Y.-Y.L.); wcsuspock@gmail.com (W.-C.S.); 5 Graduate Institute of Color and Illumination Technology, National Taiwan University of Science and Technology, Taipei 10607, Taiwan; txlee@mail.ntust.edu.tw

**Keywords:** projection lens, augmented reality, LCoS pane, magnifying glass, waveguide

## Abstract

A projection lens with a 30-degree field of view is developed for use in augmented reality (AR) glasses, including a waveguide combiner designed for a 0.35-inch LCoS panel. The entrance pupil diameter of the lens is 14 mm and the lens has an effective focal length of 16.443 mm; an F-number of 1.175. This paper has four key issues: optical projection lens design, lens manufacturing and assembly tolerance analysis, projection lens resolution testing, and AR glasses system resolution testing of panel images projected by the projection lens. After lens manufacture, the lens was tested, achieving a central field image quality of 57 cycles/mm, an angular resolution of 33 pixels per degree (PPD), a 0.7 field image quality of 40.3 cycles/mm, and an angular resolution of 23 pixels per degree (PPD). Imaging performance testing based on a diffraction-type waveguide shows a resolution of 57 cycles/mm in the center area and an angular resolution of 33 PPD.

## 1. Introduction

Head-mounted displays (HMDs) were originally used in military training and combat aid in the 1960s, similar to pilot helmets [1,2]. After decades of development, with the continuous progress of projection display technology, innovative designs, sensors, and mechanical manufacturing technology, HMDs developed into portable devices for augmented reality (AR), virtual reality (VR), and mixed reality (MR) applications, leading to the development of various commercial products [3,4,5]. Their applications span such diverse fields as firefighting and rescue [6], medical assistance [7], clinical examinations and autopsy [8], mountain rescue [9], and molecular structure simulation display [10].

The optical structures of all HMDs can generally be categorized into five types: symmetrical optical system structures, off-axis optical system structures, geometric waveguide structures, surface relief grating waveguide structures, and volume holographic optical element (VHOE) waveguide structures. The first type (namely Type 1) is the symmetrical structure, which positions the display panel at the eyepiece focus or within the focus to create a virtual image in front of the human eye. It can be implemented as a see-through structure with simultaneous projection of external images through a beam splitter [11], or a not-see-through structure without contact with the external environment [12]. This optical system includes catadioptric structures combining refractive and reflective optical elements [13], or displays with freeform surfaces, holographic plates, and prismatic structures [14,15,16], which guide light rays into the eye. The second type (Type 2) is an off-axis optical system structure composed of eccentric refractive and reflective elements [17,18]. The third type (Type 3) is a geometric waveguide structure that utilizes the principle of total internal reflection (TIR) to ensure that the incident angle of light in the waveguide is greater than the critical angle, thereby improving optical efficiency. Its prism structure redirects the outgoing beam vertically back into the eye, thereby enhancing visibility [19,20]. The fourth type (Type 4) is a surface relief grating waveguide structure, which uses periodic one-dimensional gratings on a planar waveguide to modulate light. The diffraction efficiency of gratings is determined by structural parameters such as groove depth and tilt angle [21,22,23]. The fifth type (Type 5) is the VHOE waveguide structure, which uses the volume holographic plates on the waveguide to change the direction of light, simplify the display structure, and reduce the size of the optical system [24,25,26,27].

The projection lens is a crucial optical component of HMDs, responsible for projecting images from the display to the human eye. If the lens resolution is poor, it can adversely affect the overall optical quality of the product, including the light source, illumination, color, and, of course, the image. For instance, Pan and Hung [28] designed a projection lens for a geometric waveguide display using an OLED as the light source, with a pixel size of 15 μm, pixel count of 800 × 600, lens focal length of 29.3 mm, F/# of 3.66, entrance pupil diameter of 8 mm, maximum field of view (FOV) of 30°, MTF (30 cycles/mm) > 0.4, and optical distortion < 2.73%. Hua et al. [29,30] developed a series of related symmetrical head-mounted projective displays (HMPD). In 2008, Zhang and Hua [31] designed a polarized HMPD (p-HMPD), which uses an FLCoS (ferroelectric liquid-crystal-on-silicon) sensor, with a pixel size of 13.6 μm, pixel count of 1280 × 1024, lens focal length of 21.6 mm, F/# of 2.16, entrance pupil diameter of 10 mm, maximum FOV of 55°, MTF (37 cycles/mm) > 0.4, and optical distortion < 4.0%, and RMS spot size < 16 μm. In this work, the optical projection lens development differs from the architecture of the above literature. The proposed projection lens design can be applied in various types of HMD optical structures of Type 1, Type 3, Type 4, and Type 5 displays. However, the projection lens with a 30-degree field of view is mainly used in Type 5 volumetric holographic waveguide AR glasses. The contributions of this research are mainly in the structural design, lens manufacturing, and optical performance testing of the projection lenses. In addition to the overall study of the projection lens, we also pay special attention to the imaging quality verification of the holographic waveguide AR glasses.

## 2. Design Methods

### 2.1. Projection Lens Structure Design for AR Glasses

**Figure 1:**
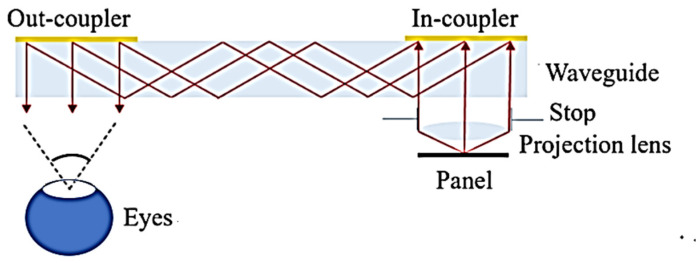
Projection lens structure design for AR glasses.

The structure of the projection lens used for the AR glasses is illustrated in Figure 1. The emissive surface of the panel is located at the focal plane of the projection lens. The primary function of the projection lens is to collimate the panel image and project it onto the in-coupler surface. A linear grating used as an input coupler guides the information light into the waveguide under total internal reflection (TIR) conditions. The information light is then guided within the waveguide. Finally, another linear grating acts as the out-coupler, directing the information light to the observer, where the image is subsequently captured by the eyes. In this situation, the final captured image consists of multiple information light beams with varying numbers of TIRs. If the information light provided by the projection lens is not well collimated, this can result in blurry or ghosting images. Therefore, the projection lens is a crucial component in a waveguide AR device. To the human eye, the projection lens structure is a magnifying glass system, and the panel image seen by the human eye will have an angle magnification effect.

### 2.2. Principle of Magnifying Glass

**Figure 2:**
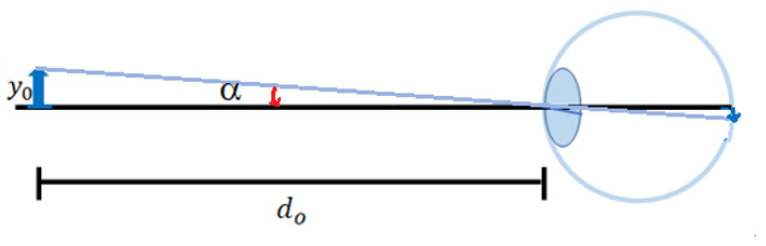
An object placed at a distinct vision distance for the human eye.

When an object with a height of *y_o_* is placed at the distinct vision distance *d_o_* (*d_o_* = −250 mm), the angle between the chief ray at the maximum height of the object and the optical axis is *α* (*α* < 0), forming an inverted reduced real image on the retina [32], as shown in Figure 2.

The relationship between the height *y_o_* of the object, the distance of distinct vision *d_o_*, and the angle

(1) $$tan\alpha =\frac{y_{o}}{d_{o}}.$$

**Figure 3:**
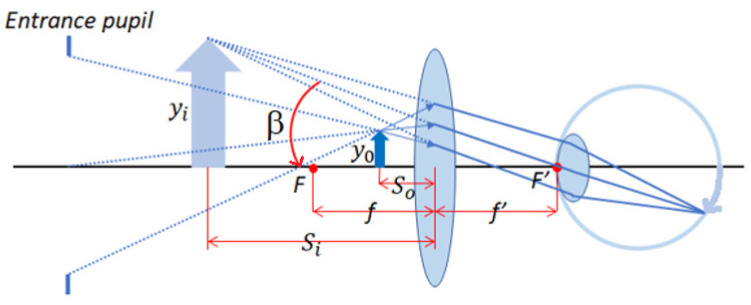
Place a magnifying glass between the object and the human eye.

If a magnifying glass is placed between an object and the human eye, and the object is located at or within the focal point of the magnifying glass, the object forms an upright magnified virtual image, and the chief ray of the highest height of this upright magnified virtual image has an angle *β* (*β* < 0) with the optical axis, as shown in Figure 3.

When an object is located at the focal point of a magnifying glass, the image of the object seen by the eyes is at infinity, which is the most comfortable state for the human eye (corrected eye). The relationship between the object height *y_o_*, the magnifying glass image focal length *f*′, and the angle *β* is shown in Equation (2).

(2) $$tan\beta =\frac{y_{o}}{f}=-\frac{y_{o}}{f^{\prime }},$$

where *f* is the object focal length of the magnifying glass, *f*′ is the image focal length of the magnifying glass; that is, *f*′ = −*f*.

The angular magnification (M_P_) of the magnifying glass can be obtained from the ratio of tanβ to tanα, as shown in Equation (3).

(3) $$M_{P}=\frac{tan\beta }{tan\alpha }=-\frac{d_{o}}{f^{\prime }}=\frac{250\;\mathrm{mm}}{f^{\prime }}$$

In the AR glasses system, the function of the projection lens is a magnifying glass system, whose angular magnification is inversely proportional to the focal length of the projection lens, and the size of the panel seen by the human eye will have an angular magnification effect.

### 2.3. Sensor and Lens Specifications

A 0.35-inch liquid crystal on silicon (LCoS) display module manufactured by Himax (Hsinchu, Taiwan) [33] is employed in the design of the 30-degree FOV projection lens. LCoS is a reflective display technology using liquid crystals to manipulate light polarization and generate an image. The specifications of the LCoS display module are listed in Table 1. The panel resolution is 1280 × 720 pixels. The active area of the panel is 7.68 mm × 4.32 mm. One half of its diagonal length is the imaging height h of the projection lens; that is, *h* = 4.406 mm.

The specifications of the projection lens are set based on the sensor specifications, with the aperture stop located on the first surface. The entrance pupil diameter is 14 mm, and the lens has a diagonal FOV of 30 degrees. A horizontal FOV of 26.29 degrees is required. The lens specifications include a F-number of 1.175, a focal length of 16.443 mm, and a maximum image height of 4.406 mm. The lens resolution is set at 35 cycles/mm, which requires angular resolution to be 20 PPD, and the specifications of the design are as shown in Table 2.

The focal length *f*′ of the projection lens is 16.443 mm, its angular magnification ratio M_P_ is 15.204. The active area of the panel is 7.68 mm × 4.32 mm, the horizontal object height, vertical object height, and diagonal object height on the panel are 3.84 mm, 2.16 mm, and 4.406 mm, respectively. If the AR glasses do not have a projection lens, then the horizontal half angle, vertical half angle, and diagonal half angle of the view of the panel seen by the human eye are *α*_||_, *α*_⊥_, and *α*, respectively. From Equation (1), we can obtain *α*_||_ = −0.880°, *α*_⊥_ = −0.495°, and *α* = −1.010°, assuming that the horizontal half angle, vertical half angle, and diagonal half angle of the panel as seen by the human eye through the projection lens are *β*_||_, *β*_⊥_, and *β*, respectively. By using Equation (3), we can obtain *β*_||_ = −13.145°, *β*_⊥_ = −7.484°, and *β* = −15.000°.

### 2.4. Tolerance Analysis

The projection lens must undergo the processes of production and manufacturing, and ultimately, testing. Therefore, tolerance analysis must be conducted to find the optimal tolerance range considering various tolerance parameters for the lens. The tolerance parameters defined in the Code V software (Version 2024.03) are shown in Table 3 [34].

Next, we define the tolerance range for each tolerance parameter. The tolerance range is the critical maximum and minimum values of the variables that can be tolerated during lens manufacturing. This range is the projection lens tolerance range used by the lens manufacturer, as shown in Table 4. The surface precision tolerance (DLF) of the lens is three to five fringes, where one fringe is 1 λ (0.586 μm); the lens thickness or air–space distance tolerance (DLT) is 0.02 mm to 0.1 mm; the lens refractive index tolerance (DLN) is 0.0002 to 0.0005; the lens dispersion with Abbe number tolerance (DLV) is 0.002 to 0.008; the angle tolerance between the wedge (TRX, TRX) and tilt (BTX, BTY) of the lens is 0.5 to 3 arcmin, which is 0.000145 to 0.000436 radian; and the decenter tolerance (DSX, DSY) for the lens assembly is 0.02 to 0.1 mm. This lens has two sets of adhesive lens groups, with adhesive tolerances (RLX, RLY) ranging from 0.01 to 0.02 mm.

## 3. Projection Lens for Design, Manufacture and Testing

The projection lens plays an important role in the AR glasses system. If the resolution of the projection lens is insufficient, the chromatic aberration or optical distortion design is defective, or the initial lens specifications do not match the panel specifications, the performance of the AR glasses will be significantly reduced, so AR glasses must match its unique lens design. This paper has four important research issues, which, respectively, discuss the projection lens design, lens manufacturing and assembly tolerance analysis, projection lens resolution testing, and AR glasses system resolution test of panel images projected by the projection lens. It is a series of complete analyses and research of the projection lenses.

### 3.1. Optical Design Data of Projection Lens

**Figure 4:**
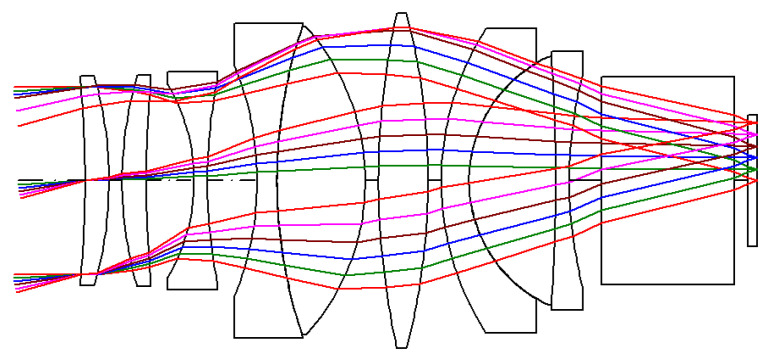
The schematic of the projection lens structure.

In the research and development of the AR glasses system, which is yet to reach the mass production stage and uses a small number of lenses, in order to reduce the manufacturing cost of lenses, we designed nine spherical lenses and improved the imaging quality of lenses with a set of doublet lenses and a set of achromatic triplet lenses. The lens design results are obtained by Code V software and optimization techniques. The projection lens has an effective focal length of 16.443 mm, and a F-number of 1.1745. The schematic of the designed projection lens structure is shown in Figure 4. The ray tracing of these different colors in the figure represents different field of view angles. To improve waveguide luminous efficiency, the entrance pupil diameter is 14 mm, and the proposed solution uses 9 lens elements to achieve the required optical specification. The projection lens design data are presented in Table 5.

**Figure 5:**
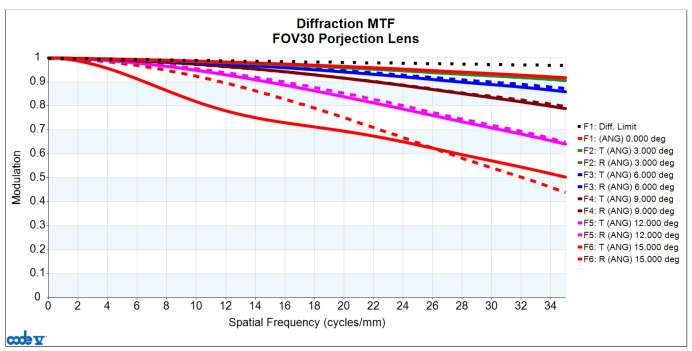
MTF plot for the designed projection lens.

The analysis of image quality includes assessments of the MTF, distortion, lateral color, and relative illumination of the lens. First, in terms of measuring MTF image quality, Figure 5 displays the MTF versus the spatial frequency curve, illustrating the relationship between variations in the continuous spatial frequency and the MTF. The horizontal axis represents spatial frequency in cycles/mm, and the vertical axis represents the MTF values. The letters “T” and “R” denote the tangential and radial directions, respectively. The lens design is targeted at a spatial frequency of 35 cycles/mm, with the minimum MTF value in this design reaching 0.440.

**Figure 6:**
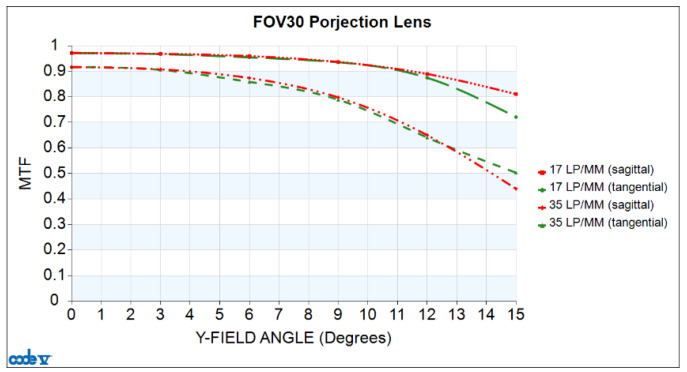
MTF versus half-field angle plotted for the projection lens.

In addition to analyzing the variations in continuous spatial frequency for the MTF, it is also necessary to assess the MTF values across the continuous field angle. Therefore, Figure 6 represents the MTF versus half-field angle curve. The horizontal axis represents the half-field angle in degrees, and the vertical axis represents MTF values. For the design with a frequency of 35 cycles/mm, the MTF values are all greater than 0.44 across the entire field of view, and the absolute difference between the RMTF and TMTF for all fields of view is less than 0.06.

**Figure 7:**
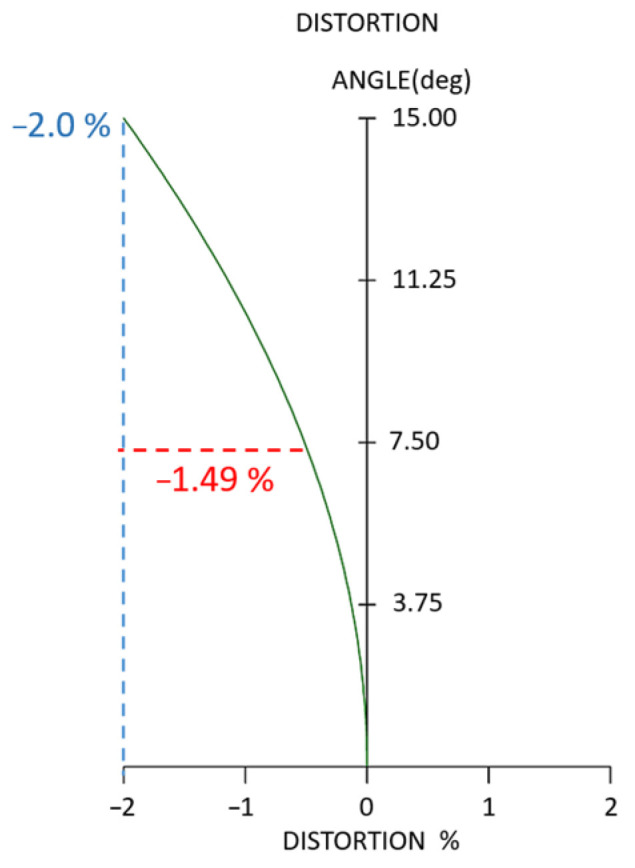
Optical distortion of the projection lens.

We present the lens design values for optical distortion and TV distortion. Figure 7 presents the optical distortion, the horizontal axis representing the percentage of optical distortion, and the vertical axis representing the half-field angle in degrees. The blue dashed line indicates optical distortion values with an absolute value of less than 2%, while the red dashed line represents the TV distortion. The TV distortion [35] is the absolute difference between the maximum and minimum optical distortion values from 0.49-field to 1.0-field and has a value of 1.49%.

**Figure 8:**
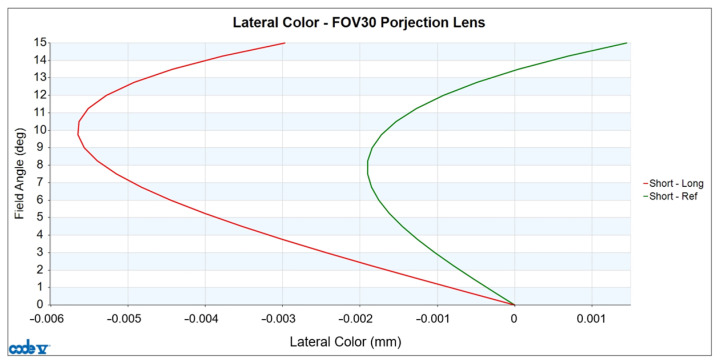
Lateral color of the projection lens.

The design values for lateral color are illustrated in Figure 8. The horizontal axis represents the differences in real image height caused by different wavelengths, measured in millimeters, while the vertical axis represents the half-field angle in degrees. The red curve shows the difference in real image height between the short wavelengths and the long wavelengths, and the green curve shows the difference in real image height between the short wavelengths and the reference wavelength. The projection lens is designed to ensure that the lateral color on the sensor is less than the size of one pixel (6 μm). This is necessary to prevent color shifts on the sensor and avoid image blurring. In this design, at 0.65 field, the maximum lateral color value, which is the difference in real image height between the short wavelengths and the long wavelengths, has an absolute value of 5.565 μm.

**Figure 9:**
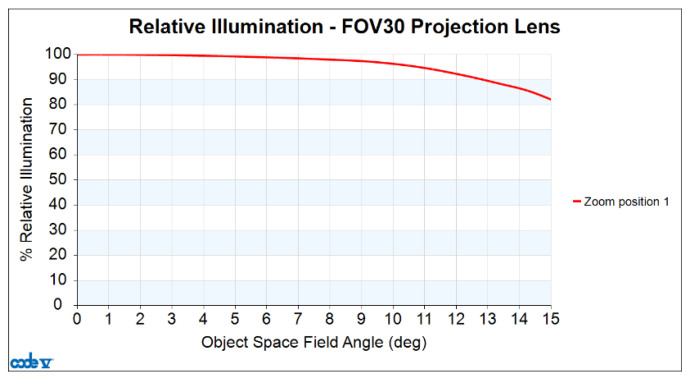
Relative illumination of the projection lens.

Finally, the values for relative illumination obtained with this design are depicted in Figure 9. The horizontal axis represents the half-field angle in degrees, and the vertical axis represents the percentage of relative illumination. The red curve illustrates the ratio of edge illuminance to central illuminance at a different half-fields angle. For this design, the relative illumination is still greater than 82.05% at the maximum half-field angle.

### 3.2. Lens Manufacturing and Tolerance Analysis

Code V optical simulation software performs lens design and tolerance analysis, and the design results are close to the actual manufacturing results. The image quality of the lens can be simulated by tolerance analysis according to the tolerance range. At this point, the designed MTF is considered ideal, but due to limitations in lens manufacturing and assembly technologies, the actual MTF after mass production may not precisely match the designed MTF. Each lens produced in large-scale manufacturing may exhibit different MTF values. Therefore, consideration must be given to how much degradation in the MTF range during lens manufacturing is acceptable. If the tolerance range is too strict, manufacturing and assembly become more challenging, leading to increased production costs. On the other hand, if the tolerance range is too loose, the MTF degradation caused by various tolerances will result in poor lens imaging quality. The establishment of acceptable tolerance ranges will also vary depending on each company’s actual manufacturing and assembly capabilities. In this design, the expected total MTF degradation due to tolerances is within 0.3. Generally, the acceptable minimum final MTF for a lens is at least 0.2. The CODE V was used to conduct a simulation of the tolerance analysis of the 30-degree FOV projection lens design at a target spatial frequency of 35 cycles/mm. Two types of tolerance analysis were conducted: centered tolerance and decentered tolerance; the centered tolerances for each surface are shown in Table 6.

The decentered tolerances include WEDGE (TRX, TRY), TILT (BTX, BTY), DEC (DLX, DLY), and ROLL (RLX, RLY). The data for these tolerances are shown in Table 7.

**Figure 10:**
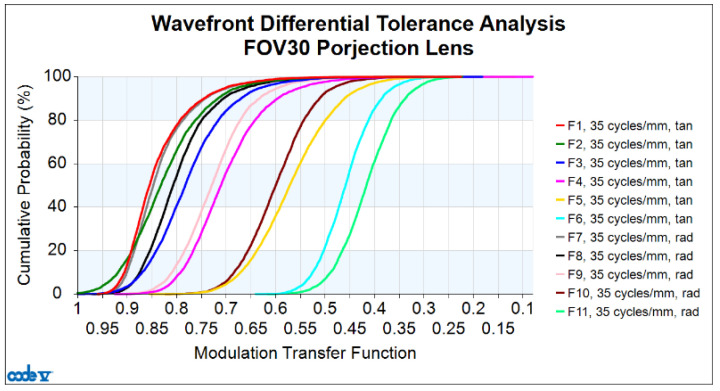
Cumulative probability versus MTF.

Finally, a comprehensive analysis incorporating all tolerance factors was carried out with the results presented in the form of a tolerance analysis curve, as shown in Figure 10. The cumulative probability is plotted on the vertical axis, while the design plus tolerance MTF is plotted on the horizontal axis. Here, TAN represents the tangential direction, and RAD represents the radial direction. For a lens manufacturing yield of 97.7%, the minimum design plus tolerance MTF for 1.0 field RAD is 0.296.

The values of the design MTF and design plus tolerance MTF for both the tangential and radial directions at a spatial frequency of 35 cycles/mm across all fields are shown in Table 8.

**Figure 11:**
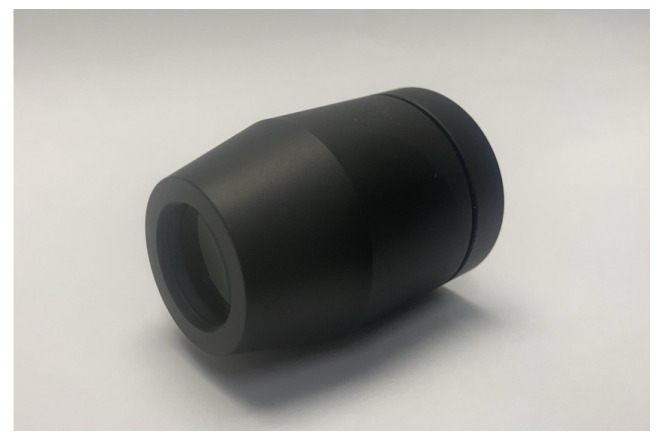
A finished projection lens with 30-degree FOV.

The tolerance compensation parameter DLZ employed to adjust the optimal imaging plane position for the best image quality has a value of 0.247 mm. The design + tolerance MTF at the 1.0 field for the radial direction reaches a minimum value of 0.296. Figure 11 shows a photo of the finished 30-degree FOV projection lens.

### 3.3. Projection Lens Testing

**Figure 12:**
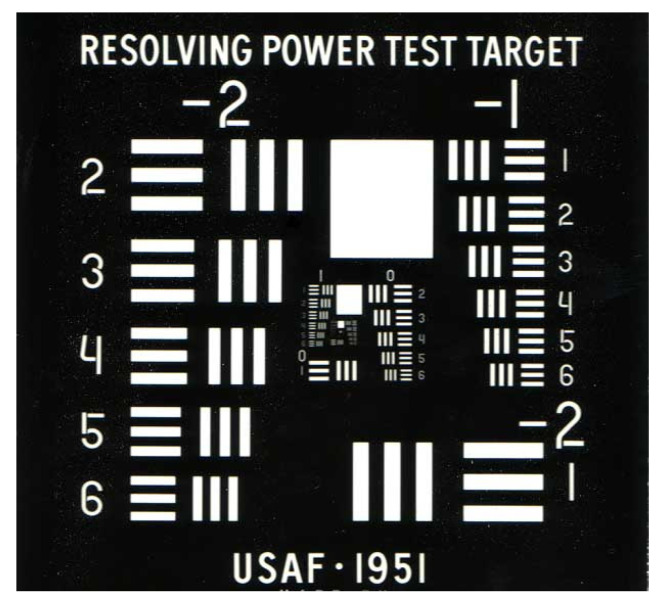
1951 USAF resolution test chart.

Lens image quality testing is performed to ensure that the images produced meet specified tolerance requirements. This study uses the back-projection detection method. The projection lens uses a white light source for lens testing. The lens is stationary and the resolution test card is located on the focal plane of the projection lens, and the white light source is located behind the resolution test card for illumination. This is a relatively simple and intuitive approach that involves visually inspecting the projected image using a test chart to evaluate projection lens resolution. Referring to the 1951 U.S. Air Force test chart resolution [36], it is a micro-optical resolution test device composed of known spatial frequency patterns, as shown in Figure 12. The spatial frequency reference chart is presented in Table 9.

**Figure 13:**
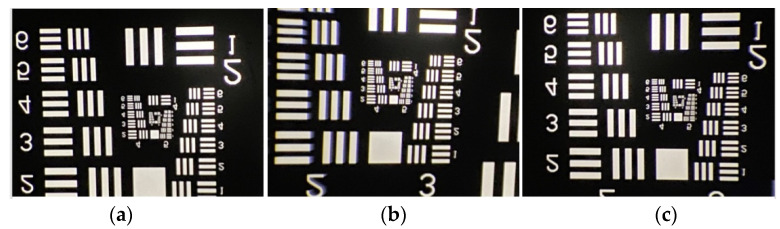
Image quality testing using the resolution test chart: (**a**) central field of view; (**b**) 0.7 field of view on the left side (−0.7 field); and (**c**) 0.7 field of view on the right side (+0.7 field).

The inverse projection testing method used for testing the lens involves placing the USAF resolution test chart on the image plane. Since the image is upside down and reverse left to right, and the effective area of the sensor is 7.68 mm × 4.32 mm, the observation range can only reach the Group 2 series and above at most, as shown in Figure 13. Figure 13a shows an image of the test chart with the center point of the test chart located at the center point of the image plane. In Figure 13b, the center point of the test chart on the image plane is shifted to the left by 3.08 mm (4.406 mm × 0.7), which indicates a field displacement of −0.7. Figure 13c shows an image in which the center point of the test chart on the image plane on the image plane is shifted 3.08 mm (4.406 mm × 0.7) to the right, a field displacement of +0.7.

**Figure 14:**
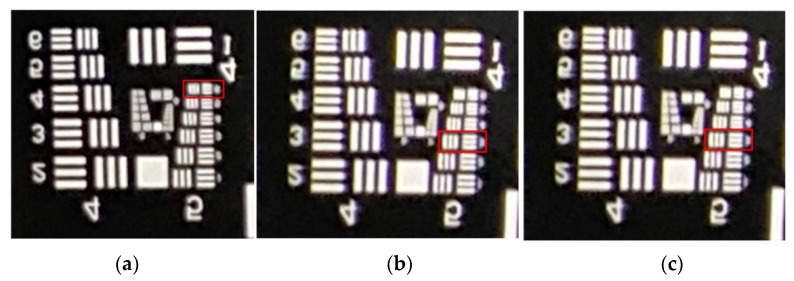
Image quality test after partial image enlargement by using resolution test chart: (**a**) central field of view; (**b**) 0.7 field of view on the left side (−0.7 field); and (**c**) 0.7 field of view on the right side (+0.7 field).

From the three images in Figure 13, we can clearly resolve graphics Element 6 of Group 4, but the details of the Group 5 graphic elements cannot be resolved because they are too small. An enlargement of the Group 4 and Group 5 graphics for the three images is shown in Figure 14. In Figure 14a, we can distinguish the details of the sixth Element details from Group 5 (57 cycles/mm). The red square in the picture represents the identifiable location. Similarly, in Figure 14b,c, we can distinguish the details of the third Element from Group 5 (40.3 cycles/mm), respectively. That is, an MTF value of 57 cycles/mm (resolution approximately 8.8 μm) is measured at the imaging center of the lens, and 40.3 cycles/mm (resolution approximately 12.4 μm) is measured at the 0.7 field of view.

Finally, the spatial frequency was input into the CODE V software for tolerance analysis, and the MTF values with the design tolerances were calculated. As can be seen in Table 10, in the central field of view at a spatial frequency of 57 cycles/mm, with an angular resolution of 33 PPD, the design tolerance MTF is 0.363. For the 0.7 field of view at a spatial frequency of 40.3 cycles/mm, with an angular resolution of 23 PPD, the TAN design tolerance MTF is 0.258, and the RAD design tolerance MTF is 0.179.

### 3.4. Performance Testing Based on a Diffraction-Type Waveguide

**Figure 15:**
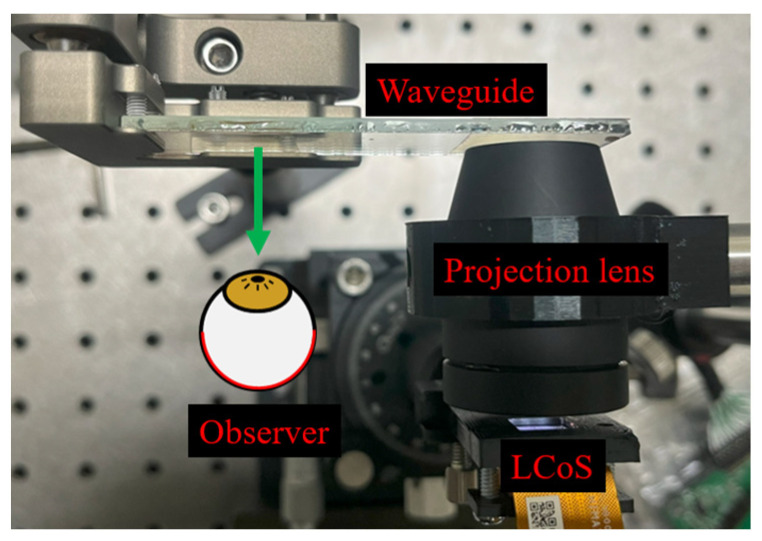
The proto-type system is employed to provide virtual images.

The image production performance of the projection lens was assessed. A VHOE-based exit pupil expander was integrated within the AR display system, as shown in Figure 15. The front-lit LCoS panel (7028FL, Himax, Hsinchu, Taiwan) was arranged at the projection lens’ object focal plane. A waveguide was utilized to guide the light information into the observer’s eye. The waveguide couplers were both VHOEs, and the structure of the out-coupler was comprised of a volume grating that is symmetrical to the in-coupler.

**Figure 16:**
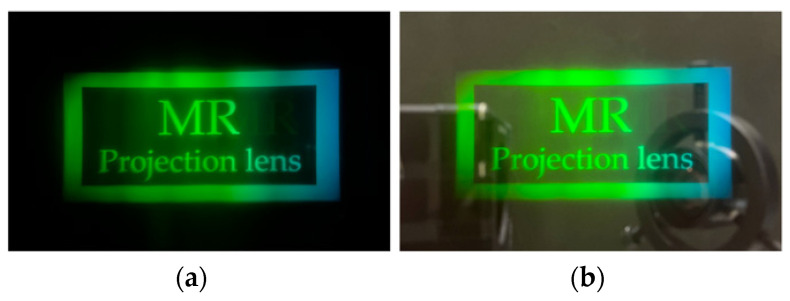
The image was captured in (**a**) dark room; (**b**) bright room.

**Figure 17:**
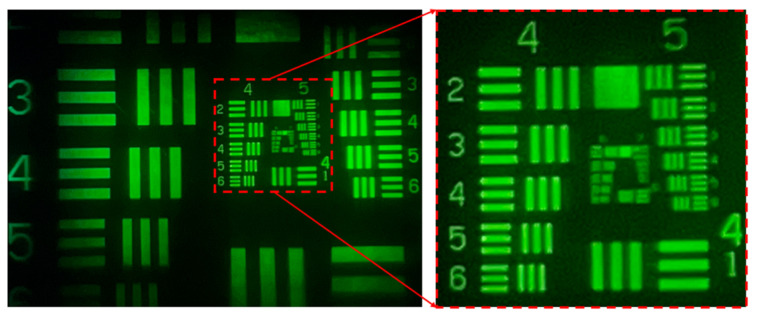
The resulting image with the 1951 USAF resolution chart proves that we can achieve a resolution of 57 cycles/mm and 33 PPD.

We can observe the image shown in Figure 16. Figure 16a shows the resulting image in a dark room; the measured FOV is 26.29 degrees. Figure 16b shows the observed image in a bright room aligns with the estimation. The image projection and the environment scene can be observed simultaneously. In our case, the in-coupler and out-coupler are VHOEs designed for green light. Consequently, the resulting image appears green since the Bragg selectivity when the front-lit LCoS panel provides a white image. Finally, the LCoS panel was replaced with a 1951 USAF resolution chart to determine the image quality. This resulting image, shown in Figure 17, Element 6 from Group 5, is visible. This shows that we can achieve an image quality of as high as 57 cycles/mm and 33 PPD.

## 4. Conclusions

This article introduces a projection lens designed for AR systems. The design of the 30-degree FOV projection lens consists of nine glass spherical lenses and two flat glass pieces. The lens design is targeted at a frequency of 35 cycles/mm, with the minimum MTF value in this design reaching 0.440, and the minimum design plus tolerance MTF at 1.0 field in the radial direction reaches a value of 0.296. For the lens test results, the resolution in the central area is 57 cycles/mm, the MTF in design + tolerance is 0.363, and the angular resolution is 33 PPD. At the 0.7 field, the resolution is 40.3 cycles/mm, the MTF in design + tolerance is 0.179, and the angular resolution is 23 PPD. Finally, the optical imaging performance test based on a diffraction-type waveguide shows that the resolution of the central region is 57 cycles/mm and the angular resolution is 33 PPD. This study successfully reaches the target value of projection lens design and can provide optical design references for potential users.

## Figures and Tables

**Table 1:** The specifications of Himax 7028FL display module.

Parameters	Specification
Manufacturer	Himax
Model No.	7028FL
Display type	LCoS
Active area diagonal	0.35 inch
Resolution	1280 × 720 pixels
Pixel pitch	6 μm × 6 μm
Active area dimensions	7.68 mm × 4.32 mm

**Table 2:** The specifications of the projection lens.

Parameters	Specification
Entrance pupil diameter	14 mm
Diagonal FOV	30°
Horizontal FOV	26.29°
Vertical FOV	14.97°
Lens Diameter	≤30 mm
Focal Length	16.443 mm
F-number	1.174
Image height	4.406 mm
MTF (35 cycles/mm)	≥0.44
LCoS protective glass material	BK7
LCoS protective glass thickness	0.7 mm
PBS material	NSF11_Schott
PBS thickness	10 mm

**Table 3:** Definitions of tolerance parameters.

Tolerance Caption	Definition
DLF	Test plate fit (power) in the fringes at 546.1 nm over the clear aperture
CYD	Cylinder (at 45° orientation) irregularity in the fringes at 546.1 nm over the clear aperture
CYN	Cylinder (at 0° orientation) irregularity in the fringes at 546.1 nm over the clear aperture
DLT	Change in the thickness (mm)
DLN	Change in the index of refraction
DLV	Change in V number if you were using d, F, and C wavelengths.
TRX	Total indicator run-out (mm) in X (resulting in a surface tilt) at the clear aperture
TRY	Total indicator run-out (mm) in Y (resulting in a surface tilt) at the clear aperture
BTX	Tilt (rad) in X of the group of surfaces about the pole of the first surface
BTY	Tilt (rad) in Y of the group of surfaces about the pole of the first surface
DSX	Lateral displacement (mm) of the group of surfaces in the X direction
DSY	Lateral displacement (mm) of the group of surfaces in the Y direction
RLX	Roll tolerances are a roll of a surface about the seat of another surface in the X direction
RLY	Roll tolerances are a roll of a surface about the seat of another surface in the Y direction
DLZ	Axial displacement (mm) of the surface

**Table 4:** The range of tolerance.

Tolerance Type	Minimum	Maximum
DLF (fringe)	3	5
DLT (mm)	0.02	0.1
DLN	0.0002	0.0005
DLV	0.002	0.008
TRX (arcmin)	0.5	3
TRY (arcmin)	0.5	3
BTX (arcmin)	0.5	3
BTY (arcmin)	0.5	3
DSX (mm)	0.02	0.1
DSY (mm)	0.02	0.1
RLX (mm)	0.01	0.02
RLY (mm)	0.01	

**Table 5:** Optical design data of the projection lens.

	Surface Type	Radius (mm)	Thickness (mm)	Glass	Full Aperture (mm)
Object	Sphere	Infinity	Infinity		
Stop	Sphere	−74.591	1.782	NSF57_SCHOTT	14
2	Sphere	−28.414	1		14.263
3	Sphere	28.595	1.815	NLASF46B_SCHOTT	14.376
4	Sphere	82.325	3.582		14.108
5	Sphere	−14.077	1	NSF11_SCHOTT	13.701
6	Sphere	40.051	3.733		14.864
7	Sphere	−22.672	1.5	NSF8_SCHOTT	16.434
8	Sphere	36.282	6.627	NLASF40_SCHOTT	20.838
9	Sphere	−17.178	1		21.793
10	Sphere	58.05	3.714	NLASF43_SCHOTT	23
11	Sphere	−49.158	1		22.993
12	Sphere	21.117	2.086	NSF57_SCHOTT	20.857
13	Sphere	10.252	6.52	NLASF44_SCHOTT	17.661
14	Sphere	−128.697	1	NSF57_SCHOTT	16.838
15	Sphere	32.134	2.432		15.369
16	Sphere	Infinity	10	NSF11_SCHOTT	14.14
17	Sphere	Infinity	1		9.793
18	Sphere	Infinity	0.7	BSC1_HOYA	8.973
19	Sphere	Infinity	0		8.772
Image	Sphere	Infinity	0		8.772

**Table 6:** Centered tolerance parameters.

Surface	DLF (fringe)	DLT (mm)	DLN	DLV (%)
1	3	0.1	0.0005	0.8
2	3	0.05		
3	3	0.05	0.0005	0.8
4	3	0.025		
5	3	0.025	0.0005	0.5
6	3	0.02		
7	3	0.02	0.0005	0.5
8	3	0.02	0.0005	0.6
9	3	0.075		
10	3	0.1	0.0005	0.8
11	3	0.1		
12	3	0.05	0.0005	0.5
13	3	0.1	0.0005	0.5
14	3	0.1	0.0005	0.8
15	3	0.1		
16	3	0.1	0.0005	0.8
17	3	0.1		
18	3	0.1	0.0005	0.8
19	3			

**Table 7:** Decentered tolerance parameters.

Element No.	TRX, TRY (arcmin)	BTX, BTY (arcmin)	DLX, DLY (mm)	RLX, RLY (mm)
1	3	1	0.02	
2	3	1	0.02	
3	1.5	1	0.02	
4	3			0.0182
4~5		1	0.02	
5	1.5			
6	3	1	0.02	
7	3			0.0154
7~8				0.0147
7~9		1	0.02	
8	2.7			
9	3			
10	3	1		
11	3	1		

**Table 8:** Design and tolerance MTF at spatial frequency 35 cycles/mm.

Field	Spatial Frequency (cycles/mm)	Azimuth(°)	Design MTF	Design + Tolerance MTF
0	35	Tangential	0.921	0.692
0.2	35	Tangential	0.908	0.652
0.39	35	Tangential	0.861	0.607
0.59	35	Tangential	0.786	0.534
0.79	35	Tangential	0.636	0.399
1	35	Tangential	0.506	0.344
0.2	35	Radial	0.911	0.685
0.39	35	Radial	0.874	0.643
0.59	35	Radial	0.794	0.572
0.79	35	Radial	0.648	0.456
1	35	Radial	0.439	0.296

**Table 9:** 1951 USAF resolving power test target (cycles/mm).

	Group Number											
Element	−2	−1	0	1	2	3	4	5	6	7	8	9
1	0.250	0.500	1	2	4	8	16	32	64	128	256	512
2	0.280	0.561	1.12	2.24	4.49	8.98	17.96	35.9	71.8	143.7	287.4	574.7
3	0.315	0.630	1.26	2.52	5.04	10.08	20.16	40.3	80.6	161.3	322.5	645.1
4	0.353	0.707	1.41	2.83	5.66	11.31	22.63	45.3	90.5	181	362	724.1
5	0.397	0.793	1.59	3.17	6.35	12.7	25.4	50.8	101.6	203.2	406.4	812.7
6	0.445	0.891	1.78	3.56	7.13	14.25	28.51	57	114	228.1	456.1	912.3

**Table 10:** The design and tolerance MTF of the projection lens.

Field	Spatial Frequency (cycles/mm)	Pixel Per Degree (PPD)	Azimuth (Direction)	Design MTF	Design + Tolerance MTF
0	57	33	Tangential	0.817	0.363
0.7	40.3	23	Tangential	0.434	0.258
0.7	40.3	23	Radial	0.330	0.179

## Data Availability

The authors do not have permission to share data.
